# Perspectives of Quantiferon TB Gold test among Indian practitioners: a survey

**DOI:** 10.1186/1869-5760-3-9

**Published:** 2013-01-11

**Authors:** Kalpana Babu, Mariamma Philips, Doddaballapur Krishnaswamy Subbakrishna

**Affiliations:** 1 Vittala International Institute of Ophthalmology & Prabha Eye Clinic and Research Center, 504, 40th Cross, Jayanagar 8th Block, Bangalore, 560070, India; 2 Department of Biostatistics, NIMHANS, Bangalore, 560029, India

**Keywords:** Quantiferon TB Gold test, India, Tuberculosis, Interferon gamma release assays

## Abstract

**Background:**

The aim of this study is to determine the preferences and perspectives regarding the Quantiferon TB Gold test for the diagnosis of tuberculosis (TB) in India. A survey was distributed among 46 uveitis specialists, rheumatologists, and pulmonologists with a minimum of 2 years experience in the management of tuberculosis, in order to restrict the respondents to specialists who have used this test in their practice in the diagnosis of tuberculosis. Topics included demographics, usage, logistics, effectiveness, and preferences related to the Quantiferon TB Gold test.

**Results:**

Among the 37 responders, there were 19 uveitis specialists, 9 rheumatologists, and 9 pulmonologists with the majority having more than 7 years of experience in treating tuberculosis. Latent TB was the most common type of tuberculosis reported by 81% of the responders. Although 92% agree that Quantiferon TB Gold assay is used for the diagnosis of latent TB, only 32% use this test always in their practice. Limiting factors include the higher cost (35.14%), limited data from countries endemic for TB and hence limited interpretation of results (32.43%), the inability to differentiate active and latent TB (32.43%), and technical issues related to the test (18.92%). A combination of the Mantoux test and Quantiferon TB Gold test was the preferred test for investigation in 51% of the responders rather than solo tests.

**Conclusions:**

Within this group of specialists dealing with different forms of tuberculosis, perspectives of this test and preferences are many. The increased cost and limited data from India with respect to interpretation of the results are the most common limiting factors in using this test.

## Background

Interferon gamma release assays (IGRAs) are new immune-based *in vitro* tests for detecting *Mycobacterium tuberculosis* (MTb) infection [1,2]. They are based on the detection of interferon gamma (IFN-γ) released by sensitized T cells on stimulation with very specific antigens, early secretory antigen target-6 and culture filtrate protein-10, both derived from a very specific region of MTb, the region of difference 1 (RD1). This segment (RD1) is deleted from all strains of BCG and the majority of environmental mycobacteria (except *Mycobacterium kansasii*[3], *Mycobacterium szulgai*, *Mycobacterium marinum*, *Mycobacterium flavescens*, and *Mycobacterium gastrii*). The two IGRAs that are available commercially include the T-Spot TB test (based on the ELISpot technology to directly count the number of IFN-γ-secreting T cells) and Quantiferon TB Gold In-Tube test (based on the ELISA technology, which measures the concentration of IFN-γ secretion; Cellestis Limited, Carnegie, Victoria, Australia). As these tests are more popular in the developed countries [4-9], their utility in a TB-endemic country like India is not clear. This is due to the limited data available on the utility of these tests from this part of the world [10-14]. Quantiferon TB Gold and Quantiferon TB Gold In-Tube tests are now available in India. The tests are being incorporated into practice as a result of their success in the developed countries. The aim of this survey is to determine the preferences and perspectives regarding the Quantiferon TB Gold test for the diagnosis of tuberculosis (TB) in India.

## Results

### Type of tuberculosis seen

Active TB was most frequently seen by pulmonologists (100%) and rarely by ophthalmologists (73.7%) and rheumatologists (89.8%). Latent TB was most frequently seen by ophthalmologists (94.7%) and rheumatologists (77.8%). This was clinically significant with a *p* value of 0.001 (Pearson chi-square test).

### Usage of this test

Although 92% agree that Quantiferon TB Gold assay is used for the diagnosis of latent TB, only 59% of respondents use this test routinely in their practice. This is slightly less than 62%, who use Mantoux test routinely in their practice. Ophthalmologists and rheumatologists use this test more often than pulmonologists (*p* = 0.024). Mantoux test is done routinely on all patients with suspected tuberculosis by ophthalmologists and rheumatologists in contrast to pulmonologists (*p* = 0.001). A Mantoux test is ‘always’ performed prior to starting immunosuppressants by 51% of respondents. This is in contrast to only 38% who always perform Quantiferon TB Gold test prior to starting immunosuppressants. Forty-six percent rarely treat a patient of suspected TB on the basis of a positive Mantoux test alone. This is similar to 43% who rarely treat a patient with suspected TB on the basis of a positive Quantiferon TB Gold test. Fifty percent of ophthalmologists and rheumatologists sometimes treat a patient with suspected tuberculosis on the basis of Quantiferon TB Gold test alone in comparison to 11.1% of pulmonologists (*p* = 0.049). Sixty-two percent of the respondents rated their experience with Quantiferon TB Gold test for suspected TB in India non-comparable with the results projected by the developed world. The preferred test for investigation in suspected tuberculosis is a combination of Mantoux test and Quantiferon TB Gold test in 51% of the responders in contrast to solo tests.

### Technical and logistic issues pertaining to the test

The test was not available in their own center in 81% of the respondents and had to be referred to a local laboratory in their own city (70%) or a laboratory outside the city (24%). The remaining (5%) was not sure where the sample is sent. In 65%, the cost of this test was equal to or more than Rs. 2,000/− (i.e., around $50). When asked regarding the frequency of ‘indeterminate’ results (always, sometimes, or never), the majority (76%) voted for ‘sometimes.’ When asked, ‘In what categories did they see an indeterminate result,’ the most common answer (86%) was ‘not sure’.

### Limitations of the test

The most common limitation of this test was the increased cost of the test (35.14%). Of the responders, 35.14% felt there was lack of enough data available from countries like India, which is endemic for TB, and hence the interpretation of the result was difficult; 18.92% felt that the test could not differentiate between active and latent TB infection; 18.92% felt technical errors while transportation or storage, poor quality control in different laboratories, and non-availability of the test in many areas were limitations of the test; 13.51% felt the test is mainly for latent TB and not active TB; 8.1% felt this test was no different from Mantoux test; 2.7% felt that this test cannot be used for monitoring treatment; and 2.7% felt that it is difficult to predict who will progress to active TB.

Results of the survey questionnaire in the three groups of responders are compiled in Table 1.

**Table 1:** Responses to the survey questions in the three groups

	**Question**	**Answer**	**Ophthalmology *N *(%)**	**Pulmonology *N *(%)**	**Rheumatology *N *(%)**	**Total *N *(%)**	**Pearson chi-square test**
1	Do you see active TB routinely in your practice	Frequently	5 (26.3)	9 (100)	1 (11.1)	15 (40.5)	0.001
		Rarely	14 (73.7)	0	8 (88.9)	22 (59.5)	
2	Do you see latent TB routinely in your practice	Frequently	18 (94.7)	5 (55.6)	7 (77.8)	30 (81.1)	
		Rarely	1 (5.3)	4 (44.4)	2 (22.2)	7 (18.9)	
3	Does your center do the Quantiferon TB Gold test in your own laboratory	No	14 (73.7)	9 (100)	7 (77.8)	30 (81.1)	
		Yes	5 (26.3)	0 (0)	2 (22.2)	7 (18.9)	
4	If your center does not do this test, is the test readily accessible to you	No	0 (0)	0 (0)	2 (22.2)	2 (5.4)	
		Yes	19 (100)	9 (100)	7 (77.8)	35 (94.6)	
5	Do you send the blood sample for this test to a laboratory outside your city	No	13 (68.4)	7 (77.8)	6 (66.7)	26 (70.3)	
		Not sure	1 (5.3)	0 (0)	1 (11.1)	2 (5.4)	
		Yes	5 (26.3)	2 (22.2)	2 (22.2)	9 (24.3)	
6	What is the approximate cost of the test to your patient	Above Rs. 2,000/−	12 (63.2)	6 (66.7)	6 (66.7)	24 (64.9)	
		Below Rs. 1,000/−	0 (0)	1 (11.1)	0 (0)	1 (2.7)	
		Between Rs. 1,000/− and Rs. 2,000/−	7 (36.8)	2 (22.2)	3 (33.3)	12 (32.4)	
7	Do you do a Mantoux test routinely on all patients with suspected TB	Always	19 (100)	1 (11.1)	3 (33.3)	23 (62.2)	0.001
		Sometimes	0 (0)	8 (88.9)	6 (66.7)	14 (37.8)	
		Never	0 (0)	0 (0)	0 (0)	0 (0)	
8	Do you consider Mantoux test a criterion for starting a patient on ATT in suspected TB	Always	7 (36.8)	0 (0)	1 (11.1)	8 (21.6)	
		Never	2 (10.5)	2 (22.2)	3 (33.3)	7 (18.9)	
		Sometimes	10 (52.6)	7 (77.8)	5 (55.6)	22 (59.5)	
9	Do you use Quantiferon TB Gold test in your practice	Never	1 (5.3)	2 (22.2)	0 (0)	3 (8.1)	
		Routinely	8 (42.1)	0 (0)	4 (44.4)	12 (32.4)	
		Sometimes	10 (52.6)	7 (77.8)	5 (55.6)	22 (59.5)	
10	How often do you get indeterminate as a result in the Quantiferon TB Gold test	Always	1 (5.3)	0 (0)	0 (0)	1 (2.7)	
		Never	5 (26.3)	3 (33.3)	0 (0)	8 (21.6)	
		Sometimes	13 (68.4)	6 (66.7)	9 (100)	28 (75.7)	
11	In what categories do you see indeterminate result in the Quantiferon TB Gold test (can tick >1 option)	Age < 18 years	1 (5.3)	1 (11.1)	0 (0)	2 (5.4)	
		Age > 60 years	0 (0)	0 (0)	1 (11.1)	1 (2.7)	
		Between ages 18 to 60 years	3 (15.8)	0 (0)	0 (0)	3 (8.1)	
		Not sure	15 (78.9)	8 (88.9)	8 (88.9)	31 (83.8)	
11	Do you perform Mantoux test routinely in all patients prior to starting immunosuppressants (not biologics)	Always	17 (89.5)	2 (22.2)	0 (0)	19 (51.4)	
		Never	2 (10.5)	3 (33.3)	5 (55.6)	10 (27)	
		Sometimes	0 (0)	4 (44.4)	4 (44.4)	8 (21.6)	
12	Do you perform Quantiferon TB Gold test routinely in all patients prior to starting immunosuppressants (not biologics)	Always	3 (15.8)	0 (0)	0 (0)	3 (8.1)	
		Never	6 (31.6)	6 (66.7)	5 (55.6)	17 (45.9)	
		Sometimes	10 (52.6)	3 (33.3)	4 (44.4)	17 (45.9)	
13	Your preferred investigation in suspected TB in your subset of patients	Both	15 (78.9)	2 (22.2)	2 (22.2)	19 (51.4)	
		Mantoux test	3 (15.8)	3 (33.3)	3 (33.3)	9 (24.3)	
		None	1 (5.3)	3 (33.3)	2 (22.2)	6 (16.2)	
		Quantiferon TB Gold test	0 (0)	1 (11.1)	2 (22.2)	3 (8.1)	
14	Do you treat a patient with suspected TB on the basis of a positive Quantiferon TB Gold test alone	Frequently	3 (15.8)	1 (11.1)	2 (22.2)	6 (16.2)	
		Rarely	7 (36.8)	7 (77.8)	2 (22.2)	16 (43.2)	
		Sometimes	9 (47.4)	1 (11.1)	5 (55.6)	15 (40.5)	
15	Do you treat a patient with suspected TB on the basis of a positive Mantoux test alone	Frequently	7 (36.8)	0 (0)	1 (11.1)	8 (21.6)	
		Rarely	5 (26.3)	8 (88.9)	4 (44.4)	17 (45.9)	
		Sometimes	7 (36.8)	1 (11.1)	4 (44.4)	12 (32.4)	
16	In your experience, how do you rate the results of Quantiferon TB Gold test for suspected TB in India with the results projected by the developed world	Comparable	10 (52.6)	2 (22.2)	2 (22.2)	14 (37.8)	0.163
		Non-comparable	9 (47.4)	7 (77.8)	7 (77.8)	23 (62.2)	
17	In your opinion, the limitation with Quantiferon TB Gold test is						

## Discussion

From this study, it is quite possible that there is a difference in the perception of Quantiferon TB Gold test by ophthalmologists, rheumatologists, and pulmonologists. This is probably due to the fact that ophthalmologists and rheumatologists see more of latent TB in their practice and depend on indirect evidences like Mantoux test and Quantiferon TB Gold test for a diagnosis, while the pulmonologists see active TB more often and depend on direct evidences like biopsy or culture for a diagnosis of TB and hence rarely use Mantoux test and Quantiferon TB Gold test in their practices.

Regarding the availability, this test is available in major cities in India. Both the second generation (Quantiferon TB Gold) and the third generation (Quantiferon TB Gold In-Tube) are available in India. Respondents who were using the second-generation test could have shifted over to the third generation in the course of time. As many of the respondents were not sure regarding the generation of the test used, this survey could not reveal the test used. This may have altered the overall perception of this test to a small extent. We do understand that the indeterminate results and false positives are lesser in the third-generation tests. However, this survey can be used as a platform to plan prospective studies in future.

High cost is a key issue. This is one of the most important limiting factors in a developing country like India. Thus, it is not feasible for individual laboratories to do this test unlike the more cost-effective Mantoux test. As a result, blood samples need to be transported to the laboratories. Quality control becomes an important factor for accurate results.

From the survey results, it is noted that less than 50% of the respondents base their treatment on the basis of a positive Mantoux test or positive Quantiferon TB Gold test alone as of now. This is probably due to the high prevalence of latent infection in an endemic country like ours. Unlike the developed world, we do not have enough evidence regarding the interpretation of this test in TB-endemic areas. As a result, its usefulness over Mantoux test in such a scenario is not very clear. In such situations, a combination of Mantoux test and Quantiferon TB Gold test was more preferred than a solo investigation as there is no gold standard for latent tuberculosis.

This survey has many limitations. Firstly, the small sample size raises questions of whether the responses collected reflect actual practice patterns of specialists in the rest of India. This population was chosen despite its small size to elicit responses from only those specialists who have used this test in the management of TB. Secondly, practice patterns regarding Mantoux and Quantiferon TB Gold may differ between individuals and hence are not uniform. This may affect the overall perception of this test to a small extent. Thirdly, this survey did not get any information regarding the generation of test used and the usefulness of this test in children and immunocompromised patients due to HIV. This is due to the limited experience available on the usefulness of this test in this subset of patients. Lastly, as only Quantiferon TB Gold and the In-Tube assays are available in India, it is difficult to get any data regarding the other IGRA test (using the ELISpot technology) from a TB-endemic country like India.

## Conclusions

From this survey, it is quite possible that there is a difference in the perception of QuantiFeron TB Gold test by ophthalmologists, rheumatologists, and pulmonologists. The increased cost and limited data from India with respect to interpretation of the results are the most common limiting factors in using this test. Even with the above limitations, this survey data can help researchers plan prospective studies regarding the usefulness of this test in TB-endemic areas in the future.

## Methods

### Survey population

Nineteen uveitis specialists all over India, belonging to the Uveitis Society of India, with experience in dealing with patients with tuberculosis and who have used this test since the last 2 years took the survey. Nine pulmonologists and nine rheumatologists with at least 2 years experience in using this test for TB also took the survey. This population was chosen despite its small size to elicit responses from only those specialists who have used this test in the management of TB.

### Survey

The survey included questions related to the type of tuberculosis seen in their practice, usage of this test in their practice, their preferences for the basis of treatment and technical and logistic issues pertaining to the test, and the limitations of this test. The respondents were asked to rate as ‘always, sometimes, or never.’ In the section on the type of tuberculosis and the preferences for the basis of treatment, the respondents were asked to rate as ‘frequent, sometimes, and rarely.’ The survey was sent online (Google) to all the respondents.

### Statistical methods

All of the statistical analysis was done using SPSS software (version 15, Chicago, IL, USA). Both descriptive analysis and independent variables were done using the chi-square test, and all tests were done at 5% level of significance.

## Competing interests

The authors declare that they have no competing interests.

## Authors' contributions

KB conceived the study, participated in its design and coordination, and drafted the manuscript. MP and DKS designed the study and performed the statistical analysis. All authors read and approved the final manuscript.
